# Hemolytic disease of the fetus and newborn: Current trends and perspectives

**DOI:** 10.4103/0973-6247.75963

**Published:** 2011-01

**Authors:** Sabita Basu, Ravneet Kaur, Gagandeep Kaur

**Affiliations:** *Department of Transfusion Medicine, Government Medical College and Hospital, Sector -32, Chandigarh, India*

**Keywords:** Newborn hemolytic disease, red cell alloimmunisation, antenatal antibody screening

## Abstract

The spectrum of hemolytic disease of the newborn has changed over the last few decades. With the implementation of Rhesus D immunoprophylaxis, hemolytic disease due to ABO incompatibility and other alloantibodies has now emerged as major causes of this condition. Though in developing countries, anti D is still a common antibody in pregnant women, many Asian countries have identified alloantibodies other than anti D as a cause of moderate-severe hemolytic disease. The most concerned fact is that, some of these have been described in Rh D positive women. It appears that universal antenatal screening in all pregnant women needs to be initiated, since Rh D positive women are just as likely as D negative women to form alloantibodies. Many developed nations have national screening programs for pregnant women. This is necessary to ensure timely availability of antigen negative blood and reduce effects on the newborn. Although universal screening seems justified, the cost and infrastructure required would be immense. Developing countries and under resourced nations need to consider universal antenatal screening and frame guidelines accordingly.

## Introduction

Hemolytic disease of the fetus and newborn (HDFN) is a condition in which transplacental passage of maternal antibodies results in immune hemolysis of fetal / neonatal red cells. The implicated antibodies could be naturally occurring (anti A, anti B) or immune antibodies which develop following a sensitizing event like transfusion or pregnancy. The hemolytic process may result in anemia or hyperbilirubinemia or both; thereby affecting fetal / neonatal morbidity and mortality.

Before the discovery of the Rhesus immunoglobulin (Rh IG), HDFN due to anti D was a significant cause of perinatal mortality. Administration of Rh IG to Rh (D) negative women during pregnancy and shortly after the birth of D positive infants has reduced the incidence of Rh D hemolytic disease.[1] ABO incompatibility is now the single largest cause of HDFN in the western world.[2] Consequent to the introduction of routine Rh IG immunoprophylaxis; alloantibodies other than anti D have emerged as an important cause of HDFN and are now responsible for greater proportion of these cases.[3] Timely detection and close follow up of this condition is necessary to reduce harmful effects on the newborn. Transfusion services play a vital role in the antenatal detection, monitoring and providing transfusion support to such cases.

## Historical overview

Thirty years ago, HDFN was almost synonymous with Rh D alloimmunization and was a common neonatal problem. The introduction of postnatal immunoprophylaxis in 1970 reduced the incidence of maternal D alloimmunization from 14% to 1-2%. Subsequently, antenatal immunoprophylaxis was also started which further reduced Rh D alloimmunization to 0.1%.[4] In the western world, ABO incompatibility is now the single largest cause of HDFN. However in many developing nations, anti D is still one of the common antibodies found in pregnant women. Besides the anti D alloantibody, moderate-severe HDFN cases attributed to other alloantibodies have been described from Asian countries in the last decade.[5,6] In addition, new treatment modalities particularly improved phototherapy and use of intravenous immunoglobulin (IVIG) have altered the clinical course of the disease.[7]

## Spectrum of hemolytic disease of newborn

Rh D- hemolytic disease, ABO- hemolytic disease and hemolytic disease due to alloantibodies other than anti D comprise the complete spectrum of HDFN.

### Rh D hemolytic disease

This condition is still commonly seen in many developing countries including India and it is likely that inadequate prenatal care or failure to administer Rh IG is responsible for this. Studies have shown that Rh (D) positive women are just as likely as D negative women to form alloantibodies. In our experience, maternal anti D alloantibody and feto-maternal ABO incompatibility are the two major causes of HDFN. However, occasional cases of severe HDFN due to non ABO, non Rh D antigens do occur.[8]

There can be considerable variation in the clinical severity of Rh hemolytic disease though direct antiglobulin test (DAT) is usually positive. Though it is generally believed that women who type serologically as Rh (D) positive have no risk for Rh D hemolytic disease, rare cases of anti D isoimmunization have been described in Rh D positive pregnant women. It appears that most of these are weak D individuals (partial D VI).[9]

### ABO hemolytic disease

With routine immunoprophylaxis with Rh IG, ABO incompatibility is now the single largest cause of HDFN in the western world. ABO hemolytic disease occurs almost exclusively in infants of blood group A or B born to O group mothers, because Ig G anti A, anti B, occur more commonly in group O than group A or B individuals. However, rare cases of ABO incompatibility have been reported in group A2 mothers with high titer anti B.[10]

Hemolytic disease of the newborn due to ABO incompatibility may also be seen in Rh (D) negative women. We encountered two infants (blood group B negative and A positive) with ABO hemolytic disease, born to group O Rh (D) negative mothers. The mother of the A positive infant did not have alloimmunization to Rh D (unpublished data). Studies have also shown that ethnic differences exist in the DAT positive prevalence amongst populations, with Asians and Blacks having higher prevalence of the DAT positive test than Caucasians.[11] The lower expression of A and B antigens on fetal red cells minimizes the feto-maternal incompatibility and hence ABO hemolytic disease is generally mild. Yet descriptions of unusually severe disease necessitating exchange transfusions and active intervention have been documented in the literature and also been reported by us.[12,13]

### Hemolytic disease due to antibodies other than anti D, anti A and anti B

The prevalence of non Rh D alloantibodies in pregnancy has been found to range from 0.15 to 1.1%.[14] In a study from Norway, about one third of the new cases of immunization to the Rh system were found in Rh (D) positive women and included anti C and anti E.[15] We have described two cases of severe HDFN, born to two Rh D positive mothers, both detected postnatally. Though the mothers were under regular antenatal supervision, the maternal alloimmunization to Rh (c) antigen went undetected.[8] At times, maternal alloimmunization could be to more than one antigen. We recently identified maternal anti C and anti E alloantibodies in a Rh (D) positive mother who delivered an infant with hemolytic disease of newborn (unpublished data).

Failure of alloantibodies of some antigen systems, eg. Le, Lu, to cause HDFN may be due to paucity of antigen sites on fetal red cells or absorption of antibodies by fetal antigen in the placenta.[16] When alloantibodies are detected in the antenatal antibody screening, the antibody must be identified, since the alloantibody specificity is a clue to its potential risk for hemolysis.

## Maternal red cell alloimmunization

Complex genetic factors influence the ability of individuals to respond to red cell antigens. Rh D is the most potent immunogen and, as little as 0.1 to 1 ml of D positive red cells can stimulate antibody production.[17] Immunization is reduced when the mother is ABO incompatible with the fetus, probably because of shortened fetal red cell life span. Besides this, the severity of HDFN is determined by the immunoglobulin subclass, amount of antibody and number of antigenic sites on the red cell.[18]

## Objectives of laboratory testing

The main objectives[19,20] of screening of all pregnant females are the following:

**ABO and Rh D typing**: It is necessary to identify the Rh D negative female. Care must be taken to use monoclonal IgM anti D grouping reagents that do not detect DVI. Also, the antiglobulin test for weak D should not be used. This is necessary to obviate the labeling of subjects with the DVI epitope and the weak D, as Rh (D) positive; since these subjects require immunoprophylaxis to prevent Rh (D) alloimmunization.

**Antibody screening and identification**: The detection and identification of antibodies gives a clue to the potential risk for HDFN and also allows sufficient time to identify cross match compatible blood for transfusion.

**Follow-up tests**: These are required to monitor the antibody strength, detect any additional antibodies if present, and determine the appropriate time for intervention.

The Rh D negative woman with an initial negative antibody screen is a candidate for Rh IG prophylaxis.

## Laboratory considerations

### Maternal testing

The blood group and Rh (D) type along with antibody screening is performed at the first prenatal visit (preferably first trimester). Monoclonal IgM anti D grouping reagents that do not detect DVI should be used and antiglobulin test for weak D should not be used. For antibody screening, the indirect antiglobulin test (IAT) using reagent red cells suspended in saline is most suitable for detection of clinically significant antibodies. Column agglutination method and solid phase methods are also suitable.[21] A validated method that detects Ig G antibodies reactive at 37°C must be used. Hence the immediate spin phase and room temperature incubation; and use of polyspecific antihuman globulin (AHG) reagent must be omitted.[22] The Ig class is established by treating maternal plasma with DTT. For patients on follow up, a change in titer by two or more dilutions is indicative of a significant change. Previously frozen serum samples must be tested in parallel to ensure that the change in titer is not due to differences in technique.[22]

Following administration of prophylactic Rh IG, anti D can be detected by IAT for up to about eight weeks. Immune anti D becomes detectable approximately four weeks after exposure to D positive cells and reaches a peak after six-eight weeks.[23] While prophylactic anti D levels fall with time, immune anti D levels usually remain stable or rise. Despite these differences, it is said that distinction between prophylactic and immune anti D may not be easy. However in our opinion, identification of immune or prophylactic anti D can be made easily, by serial determination of antibody titers along with medical history of the patient.

The Kleihauer–Betke test is a sensitive cytochemical test to identify fetal cells in maternal circulation and serves to identify subjects where additional dose of Rh IG is required. Though technically useful, we have not found it to be of much utility as it involves repeated sampling and testing to be done within 72 h of delivery, which is often not possible.

### Infant testing

The most important serologic test for the diagnosis of HDFN is the DAT with Ig G reagent. A positive DAT indicates sensitization of fetal red cells and is in itself not diagnostic of HDFN. The DAT results must be interpreted in the clinical context. Where the infant has a positive DAT with suggestive clinical findings; a red cell eluate confirms the antibody specificity, and presence of the corresponding antigen on cord cells confirms the diagnosis of HDFN. In Rh D hemolytic disease, the DAT may be strongly positive without clinical signs of disease; whereas in ABO hemolytic disease, clinical features may exist with only a weak /negative DAT.

The DAT strength does not correlate with the severity of the hemolysis and a positive DAT may invalidate the results of Rh D typing (blocked D).[24] For the resolution of the ‘blocked D’, elution technique must be performed. Elution will also be necessary when the diagnosis of HDFN is in doubt, as in rare cases of ABO incompatibility with a negative DAT.[25]

### Paternal testing

The husband’s blood group and phenotype provide useful information to predict the likelihood of a fetus carrying the relevant red cell antigen. The confirmation of the implicated antigen on the cord red cells and also on the paternal red cells confirms the diagnosis. In rare cases where HDFN is due to low prevalence antigens, maternal plasma / infant eluate is tested against paternal red cells.[26]

## Antenatal screening protocol

Antenatal screening practices vary amongst countries. In most developed countries, the increase in relative proportion of non-Rh D alloantibodies has led to the implementation of regular antenatal screening protocols.[27,28] Countries like Netherlands, Sweden have national antenatal screening programs in place for over two decades.[29,30] However, in most developing countries including India, antenatal screening is generally limited to Rh (D) negative women only.[5,31] A recent report from India stressed the need for screening Rh (D) positive women as well, after they described alloantibodies in two Rh (D) positive women.[5]

The antenatal screening protocol recommended includes testing of all pregnant women, Rh D positive or negative for ABO, Rh D type along with a red cell antibody screen at about the twelfth week of gestation.[29,32] If a clinically significant antibody is detected, monthly tests till 32 weeks and two weekly tests till term are indicated. If no alloantibody is detected in the first antenatal visit, screening should be repeated at 28-32 weeks. After this, no further testing is necessary if the antibody screens are negative.[33] For Rh (D) negative women receiving Rh IG, sampling must be done prior to the injection.

ABO incompatibility seldom causes severe hemolytic disease and routine antenatal tests to assess potency of anti A and anti B are not indicated.

## Antenatal testing and HDN status in Asian countries

Many developed nations have national antenatal screening programs such as, the Dutch screening program in Netherlands and a similar screening program in Sweden. However in developing countries, anti D continues to be a common alloantibody found in pregnant women. Failure to administer Rh IG or antenatal sensitization prior to its administration is the likely cause. Inadequate prenatal care due to unawareness and financial constraints further compound the problem. Besides anti D antibody, ABO incompatibility and other alloantibodies have been reported to cause severe HDFN in many Asian countries.

Reports from Sri Lanka, Bangladesh and India have described unusually severe ABO-HDN requiring multiple exchange transfusions.[12,34,35] In a study from Saudi Arabia, nearly one third of infants with ABO incompatibility required exchange transfusions and the use of intravenous immunoglobulin (IVIG) in such cases reduced the need for exchange transfusion.[36] Similarly IVIG has also been used in Iran in Rh D HDN to reduce the need for exchange transfusion.[37] Asian and Black infants are known to have a higher prevalence of DAT positive test than Caucasians.[11] An unusual report also describes severe ABO incompatibility in a Bangladeshi B positive mother with an A1B infant.[35]

Recent reports from some Asian countries also describe severe hemolytic disease due to alloantibodies other than anti D. A report from India describes severe hemolytic disease due to anti C and anti E in two Rh D positive women, both detected postnatally.[5] While the report from Pakistan describes two cases of hydrops fetalis due to anti K,[38] the report from Korea, Taiwan and China describe hemolytic disease due to anti C anti E, anti M, anti Jk^b^ and anti Mi.[3,6,39,40] An unusual case from Korea describes hemolytic disease due to anti Di (a) antibody which was initially missed as the panel cells lacked the antigen.[41] This emphasizes the need for proper selection of panel cells since different racial populations have different allele frequencies.

## Conclusion

The spectrum of HDFN has changed over the last few decades. Following the introduction of Rh IG, the incidence of Rh D alloimmunization in pregnancy has decreased. Consequently, hemolytic disease due to ABO incompatibility and other alloantibodies have now emerged as the major causes of HDFN. Many developed nations have implemented regular screening of all pregnant women and even have national screening programs. This is necessary to ensure timely availability of antigen negative blood and reduce effects on the newborn.

In developing countries, however, anti D is still a common antibody in pregnant women. In addition, many Asian countries have recently identified alloantibodies other than anti D as a cause of moderate-severe hemolytic disease. What is more concerning is that, some of these have been described in Rh (D) positive women. Besides, reports have also highlighted that ABO incompatibility is not always benign and may require active management.

Hence, keeping in view all of the above, universal antenatal screening in all pregnant women needs to be initiated, since Rh D positive women are just as likely as D negative women to form alloantibodies. A close follow up throughout pregnancy is required to detect irregular antibodies. Although universal screening seems justified, the cost and infrastructure required would be immense. Developing countries and under resourced nations need to consider universal antenatal screening and frame guidelines accordingly.
